# 

**Published:** 1903-02

**Authors:** 


### PERSONALS.

Dr. James Mosedale, member of the Royal College of Veterinary Surgeons, and located at Morristown, N. J., has a special department for canines and felines in his veterinary hospital.

Dr. A. I. Telmosse, of St. Andre Avellin, has removed to Hull, in the Province of Quebec, Canada.

Dr. J. T. Jones, of Atlantic City, N. J., takes a special interest in canine practice in the city by the sea.

Dr. W. H. Ridge, of Trevose, Pa., in rebuilding his home, has added a commodious office for the reception of his clientele.

Dr. W. E. A. Wyman, of Portland, Michigan, has gone to Mastin, Kansas, for a time.

Dr. I. K. Deckard, of Middletown, Pa., illustrates his stationery with an exhibition of his "Veterinary Antistitch Adhesive Plaster."

Dr. M. O'M. Knott, of Plainfield, N. J., holds the post of club veterinarian to the Union Hunt Club, of Elizabeth, N. J.

Leonard Pearson Ridge is the name of the youngest son of Dr. Wm. H. Ridge, of Trevose, Pa.

Dr. H. D. Gill, of New York, was the purchaser of a fast pacer at the Fasig-Tipton sale in New York early in December.

Dr. S. Brenton and wife, of Detroit, Michigan, were Chicago visitors in December, and the guests of Dr. A. H. Baker during a part of their stay.

Dr. Hamlet Moore, formerly of Lexington, Kentucky, has located at Danville, Kentucky.

Dr. L. N. Reefer, of Wheeling, West Virginia, is an active member of the "Elks" organization.

Dr. Leonard Pearson, of Philadelphia, State Veterinarian, was in attendance at the meeting of agriculturists in Huntington County the latter part of December.

The Journal begs to acknowledge with its appreciation the New Year greeting cards from Drs. Duncan McEachran, of Montreal, and Leonard Pearson, of Philadelphia.

Dr. M. E. Knowles, of Helena, Montana, spent two weeks in the latter part of November in Indiana, at the bedside of his sick father, whose recovery is now assured.

Dr. Ralph W. Balkam, of Hyde Park, Mass., has located at Canaan, Vermont. Dr. Balkam is a graduate of the Veterinary Department of Howard University.

Dr. B. E. Nevel, of Dover, N. J., spent several days in Philadelphia in December.

Dr. Alexander Glass, of Philadelphia, Pa., the yachtsman of the profession, spends his spare hours in fitting up his craft preparatory to going into service the coming season.

Drs. C. J. Marshall, of Philadelphia, and O. M. Norton, of Coatesville, Pa., were holiday visitors to the JOURNAL office.

Mrs. Dr. A. H. Baker, of Chicago, Ill., won a number of prizes with her cats at the Chicago pet animal show.

Dr. W. E. A. Wyman, of Portland, Michigan, was a visitor to Kansas in the month of December, doing some immunizing work among swine.

Dr. W. Horace Hoskins, of Philadelphia, spent a couple of days in December in central Virginia.

Fred. Foster, M.R.C.V.S., has been transferred from the cavalry to the artillery service in the United States Army, and is now stationed at Fort Ethan Allen, Vermont.

Dr. B. E. Nevel, of Dover, N. J., having successfully passed the State Board of Veterinary Medical Examiners in Pennsylvania, contemplates returning to his native heath, hopeful of better health than he has enjoyed in New Jersey.

Dr. S. B. Parshal, of Canfield, Ohio, offers his services as a live-stock auctioneer in a neat ad. in *The National Stockman and Farmer*, of Pittsburg, Pa.

Dr. W. V. Lusk, Veterinarian 2d Cavalry, U. S. A., is visiting his mother at Ashtabula, Ohio, where his father recently died.

Dr. M. E. Conard, of West Grove, Pa., is seriously being considered as a suitable person for filling the rôle of Secretary of Agriculture under the new administration.

Dr. J. G. Rutherford, Chief Veterinary Inspector for Canada, issued, early in January, *Bulletin No.* 9, relative to foot-and-mouth disease.

Dr. E. L. Fryer, of Blakely, Georgia, in conjunction with his practice, conducts a livery stable and deals in all stable equipments.

Dr. Leonard Pearson, State Veterinarian of Pennsylvania, was present at the inaugural ceremonies installing Hon. Samuel W. Pennypacker, Governor of Pennsylvania.

Dr. H. W. McMillen, of Brookville, Ohio, is Health Officer to that village.

Dr. D. F. Bowersox, of Aaronsburg, Pa., was attacked with pneumonia in January.

